# Supporting communication of visit information to informal caregivers: A systematic review

**DOI:** 10.1371/journal.pone.0254896

**Published:** 2021-07-22

**Authors:** Reed W. R. Bratches, Paige N. Scudder, Paul J. Barr

**Affiliations:** 1 The Dartmouth Institute for Health Policy and Clinical Practice, Geisel School of Medicine, Dartmouth College, Lebanon, New Hampshire, United States of America; 2 Center for Technology and Behavioral Health, Geisel School of Medicine, Dartmouth College, Lebanon, New Hampshire, United States of America; 3 Biomedical Libraries, Dartmouth College, Hanover, New Hampshire, United States of America; University of Maryland School of Medicine, UNITED STATES

## Abstract

**Importance:**

When caregivers cannot attend the clinic visit for the person they provide care for, patients are the predominant source of clinic visit information; however, poor patient recall inhibits the quality of information shared, resulting in poor caregiver preparedness and contributing to caregiver morbidity. Technological solutions exist to sharing clinic visit information, but their effectiveness is unclear.

**Objectives:**

To assess if and how technology is being used to connect informal caregivers to patient clinic visit information when they cannot otherwise attend, and its impact on caregiver and patient outcomes.

**Evidence review:**

MEDLINE, Cochrane, Scopus, and CINAHL were searched through 5/3/2020 with no language restrictions or limits. ClinicalTrials.gov and other reference lists were included in the search. Randomized controlled trials (RCTs) and nonrandomized trials that involved using a technological medium e.g., video or the electronic health record, to communicate visit information to a non-attending caregiver were included. Data were collected and screened using a standardized data collection form. Cochrane’s Risk of Bias 2.0 and the Newcastle-Ottawa Scale were used for RCTs and nonrandomized trials, respectively. All data were abstracted by two independent reviewers, with disagreements resolved by a third reviewer.

**Findings:**

Of 2115 studies identified in the search, four met criteria for inclusion. Two studies were randomized controlled trials and two were nonrandomized trials. All four studies found positive effects of their intervention on caregiver outcomes of interest, and three out of four studies found statistically significant improvements in key outcomes for caregivers receiving visit information. Improved outcomes included caregiver happiness, caregiver activation, caregiver preparedness, and caregiver confidence in managing patient health.

**Conclusions and relevance:**

Our review suggests that using technology to give a caregiver access to clinical visit information could be beneficial to various caregiver outcomes. There is an urgent need to address the lack of research in this area.

## Introduction

The proportion of Americans aged 65 years of age or older is expected to double in the next 20 years [1]. Among older adults in 2020, 77% are managing two or more chronic diseases; this percentage is steadily increasing [2–4]. Assistance in managing these conditions is often provided by informal caregivers: spouses, family members, or friends `who provide unpaid, at-home care to someone with whom they have a personal relationship [5,6]. In America, 53 million individuals identify as informal caregivers; 78% of those provide care for an adult over the age of 50 [7]. This increasing comorbidity of conditions that require care, with caregivers reporting their recipient has 1.7 conditions categories on average (up from 1.5 in 2015), suggests that not only are more American adults taking on the role of unpaid caregiver, but they are doing so for adult recipients who may have increasingly complex medical or support needs [7]. The number of individuals who identify as caregivers is expected to increase to 98 million by 2060 [8]. Many caregivers report that their caregiving is a positive influence on their lives, leading them to feel strong, confident, and more connected to their family members [9,10]. Additionally, a national survey of 211 informal caregivers found that positive feelings related to caregiving are associated with lower depression rates, lower caregiver burden scores, and better self-assessed health [11]. Despite the positive aspects of caregiving, providing care for older adults is stressful, and 84% of caregivers reported wanting more information to help effectively manage the care they provide [12]. Caregiving is considered a vital stress factor event, and 64% of caregivers report high or moderate stress caused by their caregiving, while up to 70% of caregivers display clinically significant symptoms of depression [13,14].

Caregiver preparedness can be defined as the perceived readiness to provide physical, emotional, and personal care [15]. Caregivers who are more prepared for caregiving demonstrate reductions in caregiving-related stress; [16] a one-unit increase in caregiver preparedness has been connected to a 17% reduction in caregiving-related stress [17]. Higher caregiver preparedness has also been linked to lower rates of healthcare utilization [18]. Caregiver preparedness is connected to the quality communication of medical information, the key source of which is the clinical visit [19]. Other sources, including the internet, can be problematic for caregivers. Seeking, accessing, and understanding health information on behalf of patients is important for caregivers to effectively provide care [20]. Caregiver electronic health (eHealth) literacy has been found to be poor; [21] and in studies of the general public low eHealth literacy has been connected to difficulties identifying quality information online as well as properly acting upon that information from eHealth sources [22,23]. Focus groups and surveys have demonstrated that the preferred source of medical information is directly from the patient’s provider, or indirectly from the provider’s notes in the patient’s electronic health record or after-visit summary [19,24–26]. Yet most information for caregivers comes directly from the patients that they care for. This, however, is problematic; patient recall is demonstrably poor, with studies showing that more than 80% of medical information is lost by the patient immediately following the visit [27]. Recall is important to maintain treatment adherence; this is especially important for those with cognitive impairments, where lower treatment adherence has been linked with greater cognitive illness severity [28].

Despite its importance, little is known about the interventions devised to directly communicate visit information with caregivers. Existing systematic reviews have found that technology-based interventions have had positive effects on caregiver outcomes; these interventions include computer-mediated caregiver skills trainings, and telephone-based caregiver support groups [29–32].

While existing systematic reviews show how technology has been successfully integrated into the caregiver experience, prior reviews do not examine how technology can be used to provide clinical information to caregivers.

Ensuring caregivers are well informed of the patient’s health issue is an essential mechanism toward better caregiving and reduced morbidity. For example, according to the Integrated Model of the Psychological Impact of Dementia Caregiving, it is hypothesized that caregiver knowledge of the illness and independence-promoting communication skills can have a positive impact on caregiver burden and related caregiver outcomes like depression, anxiety, and health services utilization [33]. Hospitals and health systems have recognized the ability for technology to impact caregiver outcomes. A systematic review published in 2014 included 33 articles of health systems adopting technology to deliver educational support to caregivers via training videos, remote consultation, problem-solving training, and for the remote monitoring of patients [34]. The authors concluded that technological solutions are beneficial to caregivers and can improve their health, quality of life, and satisfaction, but that more research is needed to identify the most effective telehealth technologies for caregivers. While technologies exist to promote the communication of clinic visit information to patients [35,36], there has been no dedicated review of these interventions, their impact on patient/caregiver outcomes, and the key ingredients of those interventions.

The aim of this systematic review was to compile currently available technology-based interventions designed to promote the communication of visit information to informal caregivers and summarize 1) the features of the interventions; and 2) their impact on informal caregiver outcomes. This review will impact current clinical practice and systems policy by understanding how interventions that communicate visit information affect patient and informal caregiver outcomes.

## Materials and methods

We adopted the Centre for Reviews and Dissemination, York University, guidance undertaking reviews in health care for the conduct of this systematic review [37]. The study protocol was not registered in an online database, but is available from the authors upon request.

### Eligibility

Studies that used technology-based interventions (I) for informal caregivers (P) to measure an effect on informal caregiver outcomes (O) were eligible for inclusion. Interventions were not required to have a comparator to be eligible for inclusion. Technologies are defined in the broadest sense: the application of scientific knowledge to practical purposes, in this case those that provide a record of clinic visit information to caregivers, and evaluated caregiver outcome measures, were included in our review. We included only interventions involving primary data collection in randomized controlled trials and nonrandomized trials. We excluded all studies where the parent is the caregiver for a child under the age of 18, as pediatric care is often different than that for adult caregivers of other adults [38]. No language exclusions were applied.

### Searching

We searched Ovid MEDLINE, CINAHL, Scopus, and Cochrane from inception to May 2020. We combined search terms related to ‘Clinic Visit Information AND ‘Caregivers’ (See S1 Appendix for full search terms). We did not limit our initial search by including terms such as “Technology” or “Digital solution”, to reduce the risk of inadvertently missing papers. We chose to manually screen titles, abstracts and full-papers that met our inclusion criteria of technology-based interventions for informal caregivers to measure an effect on informal caregiver outcomes. We did not limit our search by publication date and carried out citation index tracking using Rayyan Qatar Computing Research Institute (rayyan.ai).

### Screening and selection

All titles and abstracts were initially screened for inclusion and exclusion criteria. Abstracts that met eligibility criteria, were ambiguous, or papers that did not include an abstract, proceeded to full paper review. All screening was conducted independently by two reviewers (RWRB and PNS), with any disagreements resolved by a third member of the team (PJB). Reference lists of shortlisted full-text papers and relevant systematic reviews were searched, and additional papers identified and screened for eligibility.

### Data extraction

We extracted data relating to study aims, demographics, sample size, data collection, analysis, outcome measures, main findings, and author implications. The Data Collection Form was pilot tested on a sample of 3 papers by RWRB and PNS and refined to ensure comprehensiveness and quality. Two reviewers independently assessed risk of bias in the effect of assignment to the intervention (intention-to-treat effect) and risk of bias in the effect of adherence to the intervention (per-protocol effect) of included papers using the Cochrane Risk of Bias tool 2.0 for randomized controlled trials and the Newcastle-Ottawa Scale for nonrandomized trials (see S3 and S4 Appendices) [39,40]. By assessing risk of bias using both intention-to-treat and as-treated, the results are able to be more informative for policymakers deciding whether to recommend an intervention and individual patients and caregivers deciding on a treatment; the methods of analysis provide complementary information and reflect both real-world uptake and impact, as well as the effect under ideal conditions [41]. Analysis done via intent-to-treat estimates the effect of treatment assignment, which is more conservative and could underestimate the true effect, but reduces bias through the preservation of the randomization of study participants [42]. Per-protocol analysis better reflects the effect of strict adherence to an intervention, but as a result, provides lower-quality evidence as differences observed could be due to unmeasured confounders that are no longer randomly allocated across the groups [43].

### Data synthesis

Data were synthesized through narrative synthesis using the Cochrane methodology, adapted from guidelines described in Popey et al., but a meta-analysis was unable to be performed due to heterogeneous outcome measures [44,45]. We began by describing the included studies and grouping them by common themes. Two reviewers examined study designs, risk of bias, and study results to identify patterns to inform our within- and between- study comparisons, with attention paid to variability in outcomes, study designs, populations, interventions, and settings. The GRADE methodology was used to assess the overall quality of the evidence included in the study [46].

## Results and discussion

### Description of included studies

Out of an initial search that yielded 2115 results. After screening for duplicates, 1603 unique records underwent title and abstract screening, of which 1561 were deemed ineligible. The 42 remaining papers were screened in their entirety. Of these articles, 18 were ineligible for being opinion pieces or qualitative studies, 12 did not include information from the clinic visit, and 6 mentioned caregivers but measured no caregiver outcomes. Ultimately, four articles were list of reasons for exclusions at the stage of eligibility assessment. There was a 94.8% agreement between annotators on eligibility screening, and all disagreements were resolved.

One study was conducted in Japan [47] and the other three studies [48–50] took place in the United States. Two studies were randomized controlled trials [47,48] and two were nonrandomized trials [49,50], with sample sizes ranging from 13–252 caregivers. Hori et al. [47] randomized caregivers of patients with dementia to receive after visit communication with the patient’s clinician via video call. In Toye et al. [48], caregivers of neurology and oncology patients received three telephone calls with the provider, where they were guided through the discharge information and received a needs assessment. In Schnock et al. [49], caregivers of older adults received access to a web-based application in the patient portal that included discharge information. In Wolff et al. [50], caregivers were given access to the patient’s after-visit summary through the patient portal account. Follow-up periods varied greatly, from as little as 6 weeks to as long as 18 months. Although all interventions involved the communication of clinic visit information to caregivers, the interventions and outcomes varied across studies, prohibiting the use of meta analytic techniques to quantitatively synthesize outcomes. See Table 1 for more information.

**Table 1:** Characteristics of included studies.

Authors	Hori M, Kubota M, Ando K, Kihara T	Schnock K; Snyder J; Fuller T	Toye C; Parsons R; Slatyer S; Aoun S	Wolff JL; Darer JD; Berger A; Clarke D
Title	The effect of videophone communication (with skype and webcam) for elderly patients with dementia and their caregivers	Acute Care Patient Portal Intervention: Portal Use and Activation	Outcomes for family carers of a nurse-delivered hospital discharge intervention for older people	Inviting patients and care partners to read doctor’s notes: OpenNotes and shared access to electronic medical records
Year	2009	2019	2016	2017
Study Design	RCT	pre/post	single blind RCT	Non-randomized trial
Quality Rating Scheme for Studies and Other Evidence	2	2	2	2
Study Duration	12 weeks	18 months	6 weeks	12 months
Research Question	Do videophone interventions for patients and caregivers affect cognitive attributes of caregiver outcomes?	What is the association between acute care patient portal use and patinent/caregiver activation in a hospital setting?	How does implementing the Further Enabling Care at Home program influence the outcome for patient caregivers?	What are patients’ and care partners’ perceptions of OpenNotes, their confidence in managing aspects of patient care, and online practices?
In/Outpatient	Outpatient	Inpatient	Outpatient	Outpatient
Participants	Caregivers of patients with dementia	Caregivers of patients in a general medicine, neurology, or oncology service	Caregivers from the medical assessment unit in a tertiary hospital in Western Australia	age 18+ with shared access to their patient portal account
Number of Study Arms	2	1	2	1
Intervention	Once per week, a 30 minute clinical communication video call with the provider	Use of the web-based application in the patient portal.	Further Enabling Care at Home (FECH) Program: 3x telephone calls with the provider	Access to OpenNotes via Patient Portal
Control/Comparator	Caregivers of patients with dementia that did not receive the intervention	Pre-intervention caregivers	Caregivers who did not receive the intervention	N/A
Outcome	Happiness Visual Analog Scale	Patient activation, caregiver activation	caregiver preparedness	confidence in managing aspects of health
Measure(s)	VAS	CG-PAM	Preparedness for Caregiving Scale	Self-report
Sample Size	13	156	141	252
Principle Findings	The intervention group reported no change in VAS, while the control group reported significantly decreased VAS.	Statistically significant effects for caregivers of neurology and general medicine patients. Small caregiving n across practice groups (n = 6 in oncology) makes formal statistical comparisons difficult, but observed trends similar to PAM	FECH program improved caregiver preparedness across all domains and times by 2 points.	Care partners were more likely to communicate with providers

*RCT = Randomized Controlled Trial; VAS = Visual Analogue Scale’ PAM = Patient Activation Measure; FECH = Further Enabling Care at Home.

### Effects of interventions

#### Primary outcomes

Primary outcomes focused on the impact of the intervention on caregiver behavior and emotional affect.

*Emotional affect*. Hori et al. [47] measured caregiver happiness using a self-reported Visual Analogue Scale on a scale of 0 (not happy) to 100 (perfectly happy). Caregivers who received after visit information from the clinician via video call (n = 7) reported no change between the initial measure and the follow-up 12 weeks later (79.2 at T_1_, 79.3 at T_2_) but those in the control group (n = 6) reported a statistically significant decrease in happiness of 19.9 points at 12 weeks (71.6 at T1 to 51.7 at T_2_, p = 0.027).

*Caregiver behavior*. Toye et al. [48] reported a statistically significant 2 point increase in caregiver preparedness for the intervention group (n = 77) who received discharge information via phone call with the patients clinician, compared to the controls (n = 64), at 3 weeks (Cohen’s d = 0.43, p = 0.019).

Schnock et al. [49] compared the same caregivers (n = 156) before and after access to visit summaries in the patient portal, and reported a non-statistically significant increase in CareGiver-Patient Activation Measure at 18 months, but the degree was not reported. [51] Upon contacting the author, they reported that the small number of caregivers precluded an independent analysis of caregiver activation.

Wolff et al. [50] reported that caregivers (n = 252) given access to an after visit summary (AVS) asked more questions of the provider, compared to those who did not receive an AVS (Odds Ratio 1.74, CI 1.36, 2.23).

### Quality of design, methods, and reporting

Overall, the methodological quality for the results of the included studies was deemed to be good (see S3 Appendix for detailed risk of bias assessment).

While the two trials received scores of “Some Concerns” [47] and “Low” [48] on the Cochrane Risk of Bias Tool [39], they both reported low attrition and well-described randomization schemes. Impressive points from both studies include adherence to the intervention; both studies’ low attrition rates are due in part to the diligence of the follow-up emails and telephone calls. However, Hori et al. [47] provided little information on the effect of assignment to the intervention and their outcome measure was not validated.

The two nonrandomized trials were assessed through the Newcastle-Ottawa Scale, and both received a score of “Good”. Schnock et al. [49] used a well-developed propensity matching scheme to maintain equivalence between groups in different hospital sections, and aid the comparability of their cohorts at baseline. Wolff et al. [50] used a thorough outcome assessment that involved online usage metrics as well as message exchanges, and their large sample size allowed them to create a representative cohort. However, both studies provided limited information on the representativeness of the follow-up cohorts relative to those who received treatment.

The effect of adherence to the intervention and the effect of assignment to the intervention are unlikely to introduce bias, and we would expect the effect estimates to remain similar due to the low attrition and effective randomization schemes observed in all studies. The risk of bias in reported findings is low.

The sample size of the included studies could affect the observed outcomes, particularly the Hori et. al paper where only 13 caregivers were included [47], and Schnock et al., who reported that they were unable to determine a statistically significant measure due to the small number of caregivers in each clinical specialty [49]. The Toye and Wolff studies were adequately powered [48,50].

Overall evidence quality was determined using GRADE for both RCTs and observational studies (see Table 2). Overall, moderate quality of evidence shows that technology can benefit caregiver outcomes. The level of evidence for RCTs was downgraded due to lack of blinding in outcome assessment, and the level of evidence for observational studies was not upgraded despite one study demonstrating a large positive impact.

**Table 2:** 

No. of participants	Risk of Bias*	Inconsistency	Indirectness	Imprecision	Publication Bias	Overall Assessment
Randomized Controlled Trials						
154	Low	Not Serious	Not Serious	Not Serious	Unclear (low number of studies)	Strong for Using
Observational Studies						
408	Good	Not Serious	Not Serious	Not Serious	Unclear (low number of studies)	Weak for Using

*Cochrane ROB2.0 for RCTs, Newcastle-Ottawa Scale for observational studies; Low indicates low risk of bias, while Good indicates the studies did not exhibit concerning risk of bias.

## Discussion

### Summary of main results

In our review, only four studies examined the impact of technology-based interventions to promote the sharing of clinic visit information with caregivers of patients–vital to successful disease self-management and caregiver well-being. Included papers reported positive impacts on caregiver behavior and affect, when using technology to support caregiver access to visit information–especially when delivered by the clinician. Technological interventions show promise across a variety of domains and conditions, including dementia, neurology, oncology and aging-associated care. The general quality of the included studies and designs was good. While these studies do not allow robust conclusions to be drawn, findings are promising but more studies with larger caregiver samples are needed. Further studies are required and should include data on health outcomes for caregivers such as mental and physical well-being. Overall quality of evidence from the GRADE analysis was assessed as moderate. Thus, more research will have a large impact on our ability to understand the true effect of technological interventions on caregivers [52].

### Comparison with prior work

While there have been no prior reviews examining how technology is used to facilitate the communication of visit information, similar reviews have found technology to be effective in providing e-support, like caregiver support groups or general condition information through technology, to caregivers [30]. Our review deepens this finding by extending the provision of e-support to clinical information, and our findings demonstrate that the impacts are especially positive when a member of the clinical team is involved in the caregiver contact [47,48]. Scaling interventions that involve clinical team members contacting each caregiver may be a challenging. Sharing clinic recordings (audio or video) may be a more scalable solution, as it allows the caregivers access to the entire clinic visit and could be efficiently added to the patient portal, reducing the need for additional phone/video calls to caregivers [27,53]. Our finding of greater access to visit information and the proceeding improvements in caregiver wellbeing and confidence in disease management support the findings from other previous studies [12].

This review also supports the qualitative findings that caregivers can perform more effectively with access to direct and indirect information that is specific to their caregiving experience [19,24]. This is apparent in the findings of Hori et al. [47] where caregivers were happier, and with Toye et al. [48] who saw an increase in caregiver preparedness with access to clinic information. This increase in preparedness could be connected to the higher likelihood of caregivers asking questions when they have notes, which was found in Wolff et al. [50].

Despite the positive findings, there are too few studies to make firm conclusions or recommendations on how best to support caregivers in receiving this information. Prior reviews have also reported the need for the further research of focused on communication with caregivers [16].

### Strengths and limitations

As in any review, one limitation is the potential for our search strategy to have missed some relevant papers. Our approach regarding the exclusion of children, despite being the norm for caregiver interventions due to consent concerns [12], could have resulted in potentially useful papers not being included. Yet the experience of caregivers of children is different from that of adults; a separate review addressing communication of visit information to caregivers of children would be a helpful addition to the literature. However, we feel that this risk is reduced by our inclusive and comprehensive search strategy, which was developed by a biomedical librarian and iteratively refined with the help of outside reviewers. After an initial search with overly-restrictive criteria returned no included papers, we refined the strategy to be as sensitive as possible while capturing papers relevant to the research question. This involved using comprehensive terms and reviewing reference lists of included studies to ensure completeness.

### Implications

While patients now have access to their visit information it is critical that greater focus is placed on how to provide access to this information to the estimated 43.5 million Americans who identify as lay caregivers [50]. Such access can improve caregiver satisfaction and preparedness to provide care, both of which are associated with better health outcomes for patients and caregivers [16]. More effective caregiving could also lead to lower healthcare utilization [18]. While our review and others highlight the needs for further research in the field of caregiving, policy makers also have an important role to play. Policymakers should consider the impact of technology on caregivers in addition to patients, particularly as caregivers enter legislation and policy such as the RAISE Family Caregivers Act, which directs the creation of a national caregiver strategy [54]. The RAISE Family Caregiving Advisory Council develops and executes the national strategy, and has identified “information and education” as strategic priorities, and recent funding from the Coronavirus Aid, Relief, and Economic Securities (CARES) act has been explicitly directed to support caregiver resource consultation [55,56]. The Center for Law and Social Policy recommends that states ensure all communications with essential workers indicate and support family caregivers [57]. The results of this review indicate technology could be an effective medium to support the missions of the RAISE and CARES acts. Additionally, as telehealth becomes more widespread and accepted, the use of technology based interventions such as those outlined by Hori et al. are becoming more commonplace [47]. Understanding the benefits of these telehealth interventions, not only to patients but to caregivers, will be critical to ensuring patterns of care include the full treatment team; these benefits could also extend to caregiver outcomes as outlined in the Integrated Model of the Psychological Impact of Dementia Caregiving like depression, anxiety, and health services utilization [33].

These results are particularly germane in light of the COVID-19 pandemic. Prior to the COVID-19 pandemic, much of healthcare information was communicated in-person [58]. The rapid shift to telemedicine during the COVID-19 pandemic has led to a disruption of normal caregiving routines, and caregivers can have difficulty using telemedicine technology [59,60]. Our review indicates there are benefits to communicating information from the clinic visit to caregivers that result in improved caregiver outcomes, such as caregiver happiness and visit preparedness. While stay-at-home policies have limited access to in-person clinics, health systems should consider using video to connect with family caregivers; these policies could also provide an opportunity to include caregivers who may have otherwise been unable to attend the clinic visit.

This review provides promising beginnings that open a useful starting point for future research.

## Conclusions

Using technology to facilitate the provisioning of visit information to caregivers has the potential to increase caregiver activation and preparedness, while resulting in improved health outcomes for both caregivers and patients. The need is significant, with 21% of American’s providing informal caregiving to individuals with ever increasing medical complexity. Yet with only four studies included in our review, more research is needed to identify the optimal strategies for the provision of clinical visit information to caregivers.

## Supporting information

S1 Appendix Search strategy. (DOCX)

S2 Appendix PRISMA flow diagram. (DOCX)

S3 Appendix Newcastle-Ottawa risk of bias scale. (DOCX)

S4 Appendix Cochrane risk of bias scale. (DOCX)

S5 Appendix PRISMA statement. (DOCX)
