# Supplementary material for: Supporting communication of visit information to informal caregivers: A systematic review
Source: PLoS One. 2021 Jul 22;16(7):e0254896. doi: 10.1371/journal.pone.0254896 (PMC8297802; doi:10.1371/journal.pone.0254896)
Supplement: S1 Appendix — (DOCX) [file pone.0254896.s001.docx]

Appendix 1: Search Strategies

Search Strategies (5/3/2020)

| Medline (OVID) | |  |
| --- | --- | --- |
| # | Search | Results |
| 1 | exp telemedicine/OR exp/telecommunication/ OR exp patient portal/ OR Communication/mt | 90971 |
| 2 | exp caregiver/ | 35994 |
| 3 | (carer OR caretaker OR caregiver).ti,ab. | 32207 |
| 4 | (infan* OR newborn* OR new-born* OR perinat* OR neonat* OR baby OR baby* OR babies or toddler* OR minor OR minors* OR boy OR boys OR boyfriend OR boyhood OR girl* OR kid OR kids OR child OR child* OR children* OR schoolchild* OR schoolchild OR school child OR school child* OR adolescen* OR juvenil* OR youth* OR teen* OR "under age" OR pubescen* OR pediatric* OR paediatric* OR peadiatric* OR school* OR prematur* OR preterm*).ti,ab. OR exp pediatrics/ | 2657068 |
| 5 | ("visit summary" OR telemedicine OR telecommunication OR patient portal OR telehealth OR email OR "video conferencing" OR "discharge summary").ti,ab. | 22817 |
| 6 | 1 OR 5 | 102266 |
| 7 | 2 OR 3 | 54244 |
| 8 | 6 AND 7 | 1166 |
| 9 | 8 NOT 4 | 872 |
|  |  |  |
|  |  |  |
| CINAHL |  |  |
| # | Search |  |
| 1 | telemedicine OR telehealth OR patient portal OR communication |  |
| 2 | visit summary OR patient portal OR telecommunication |  |
| 3 | email OR video conferencing OR discharge summary |  |
| 4 | carer OR caregivers OR caretaker OR caregiver |  |
| 5 | infant OR newborn OR new-born OR neonate OR baby OR babies OR toddler OR minor OR minors OR boy OR boys OR boyfriend OR boyhood OR girl OR kid OR kids OR child OR child OR children OR schoolchild OR adolescent OR juvenile OR youth OR teen OR "under age" OR pubescent OR pediatri* OR paediatric OR peadiatric OR school |  |
| 6 | 1 AJND 2 AND 3 AND 4 |  |
| 7 | 6 NOT 5 |  |
|  |  |  |
|  |  |  |
| SCOPUS |  |  |
| # | Search |  |
| 1 | telemedicine OR telehealth OR patient portal OR visit summary OR telemedicine OR telecommunications |  |
| 2 | caregiver OR caretaker OR carer |  |
| 3 | email OR video conference OR disharge summary OR visit summary |  |
| 4 | infant OR newborn OR new-born OR neonate OR baby OR babies OR toddler OR minor OR minors OR boy OR boys OR boyfriend OR boyhood OR girl OR kid OR kids OR child OR child OR children OR schoolchild OR adolescent OR juvenile OR youth OR teen OR "under age" OR pubescent OR pediatri* OR paediatric OR peadiatric OR school |  |
| 5 | 1 AND 2 AND 3 |  |
| 6 | 5 NOT 4 |  |
|  |  |  |
| Cochrane Library | |  |
| # | Search |  |
| 1 | Telemedicine OR telecommunication OR patient portal OR communication |  |
| 2 | caregiver OR carer OR caretaker |  |
| 3 | visit summary OR summary OR discharge summary |  |
