# Supplementary material for: Supporting communication of visit information to informal caregivers: A systematic review
Source: PLoS One. 2021 Jul 22;16(7):e0254896. doi: 10.1371/journal.pone.0254896 (PMC8297802; doi:10.1371/journal.pone.0254896)
Supplement: S2 Appendix — (DOCX) [file pone.0254896.s002.docx]

Appendix 2: PRISMA Flow Diagram

0 Additional records identified through other sources (grey literature, conference abstracts, unpublished studies)

**Appendix 6**

1603 Unique records from all sources
 (duplicates removed)

1603 Records screened for eligibility via Title and Abstract

42 Full-text records assessed for eligibility

2115 Records identified through electronic database searching

Ovid Medline – 859
CINAHL – 662

SCOPUS – 594

Cochrane Library – 0

0 Ongoing studies

38 Records excluded by full-text review:

-18 were opinion pieces or qualitative studies

-12 did not include information from the visit

-6 mentioned caregivers, but did not measure caregiver outcomes

4 Studies included in narrative synthesis

4 Studies excluded from meta-analysis

No common outcomes

Unable to translate-x

0 Studies included in quantitative synthesis (meta-analysis)

1561 Records excluded by abstract & title review
