# Supplementary material for: Supporting communication of visit information to informal caregivers: A systematic review
Source: PLoS One. 2021 Jul 22;16(7):e0254896. doi: 10.1371/journal.pone.0254896 (PMC8297802; doi:10.1371/journal.pone.0254896)
Supplement: S3 Appendix — (DOCX) [file pone.0254896.s003.docx]

Appendix 3: Newcastle-Ottawa Scale

| Newcastle-Ottawa | Schnock K; Snyder J; Fuller T | Wolff JL; Darer JD; Berger A; Clarke D |
| --- | --- | --- |
|  | Acute Care Patient Portal Intervention: Portal Use and Activation | Inviting patients and care partners to read doctor's notes: OpenNotes and shared access to electronic medical records |
| Representativeness of Exposed Cohort | * | * |
| Selection of non-exposed cohort | * | * |
| Ascertainment of exposure | * | * |
| Demonstration that outcome of interest was not present at the start of study | no | * |
|  | 3 | 4 |
| Comparability of Cohorts on the basis of the design or analysis controlled for confounders | * | * |
|  | 1 | 1 |
| Assessment of Outcome | * | * |
| Was follow-up long enough for outcome to occur | * | * |
| Adequacy of follow-up cohort | no | no |
|  | 2 | 2 |
| Total | 6 | 7 |
| AHRQ Standard | GOOD | GOOD |
